# Do biofilm communities respond to the chemical signatures of fracking? A test involving streams in North-central Arkansas

**DOI:** 10.1186/s12866-017-0926-5

**Published:** 2017-02-03

**Authors:** Wilson H. Johnson, Marlis R. Douglas, Jeffrey A. Lewis, Tara N. Stuecker, Franck G. Carbonero, Bradley J. Austin, Michelle A. Evans-White, Sally A. Entrekin, Michael E. Douglas

**Affiliations:** 1 Department of Biological Sciences, University of Arkansas, Fayetteville, AR USA; 2 Department of Food Sciences, University of Arkansas, Fayetteville, AR USA; 3 Department of Biology, University of Central Arkansas, Conway, AR 72035 USA

**Keywords:** 16S ribosomal RNA, Anthropogenic impacts, Bioindicators, Fayetteville shale, Groundwater, Microbiome

## Abstract

**Background:**

Unconventional natural gas (UNG) extraction (fracking) is ongoing in 29 North American shale basins (20 states), with ~6000 wells found within the Fayetteville shale (north-central Arkansas). If the chemical signature of fracking is detectable in streams, it can be employed to bookmark potential impacts. We evaluated benthic biofilm community composition as a proxy for stream chemistry so as to segregate anthropogenic signatures in eight Arkansas River catchments. In doing so, we tested the hypothesis that fracking characteristics in study streams are statistically distinguishable from those produced by agriculture or urbanization.

**Results:**

Four tributary catchments had UNG-wells significantly more dense and near to our sampling sites and were grouped as ‘potentially-impacted catchment zones’ (PICZ). Four others were characterized by significantly larger forested area with greater slope and elevation but reduced pasture, and were classified as ‘minimally-impacted’ (MICZ). Overall, 46 bacterial phyla/141 classes were identified, with 24 phyla (52%) and 54 classes (38%) across all samples. PICZ-sites were ecologically more variable than MICZ-sites, with significantly greater nutrient levels (total nitrogen, total phosphorous), and elevated Cyanobacteria as bioindicators that tracked these conditions. PICZ-sites also exhibited elevated conductance (a correlate of increased ion concentration) and depressed salt-intolerant Spartobacteria, suggesting the presence of brine as a fracking effect. Biofilm communities at PICZ-sites were significantly less variable than those at MICZ-sites.

**Conclusions:**

Study streams differed by Group according to morphology, land use, and water chemistry but not in biofilm community structure. Those at PICZ-sites covaried according to anthropogenic impact, and were qualitatively similar to communities found at sites disturbed by fracking. The hypothesis that fracking signatures in study streams are distinguishable from those produced by other anthropogenic effects was statistically rejected. Instead, alterations in biofilm community composition, as induced by fracking, may be less specific than initially predicted, and thus more easily confounded by agriculture and urbanization effects (among others). Study streams must be carefully categorized with regard to the magnitude and extent of anthropogenic impacts. They must also be segregated with statistical confidence (as herein) before fracking impacts are monitored.

## Background

**Fig. 1:**
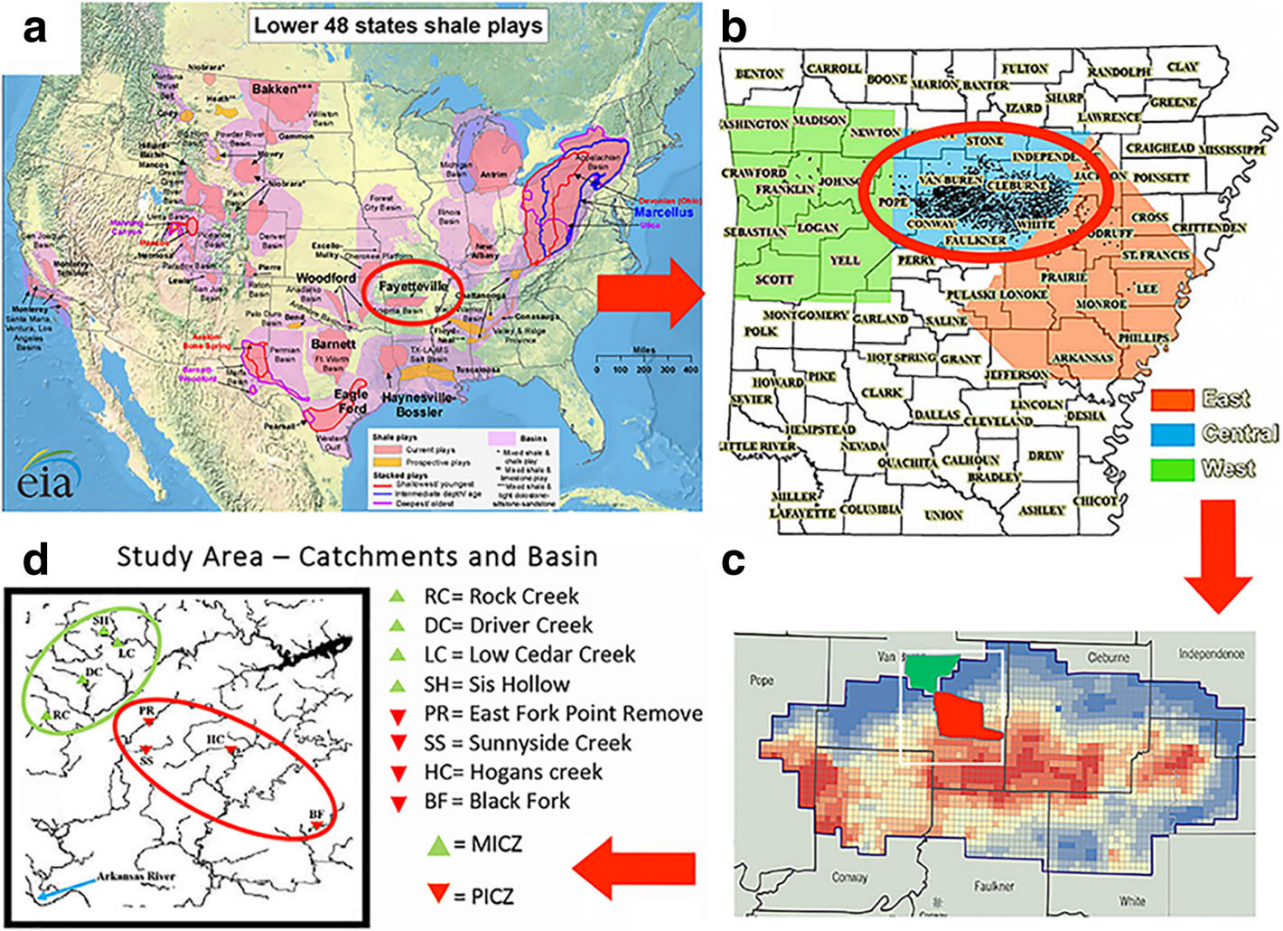
**a** Map depicting shale plays located in the United States, with the Fayetteville Shale circled in *red* [44]; (**b**) Map of Arkansas counties showing the topographic location of the Fayetteville shale, with eastern (*red*), central (*blue*), and western (*green*) sections highlighted. The study region is *circled in red*, with closed *black circles* designating the locations of unconventional natural gas (UNG) well sites; **c** Close-up of the northern Arkansas counties within which the Fayetteville shale is distributed. The region in *red* is designated as the ‘potentially impacted catchment zone’ (=PICZ), a region with high UNG well density, whereas the region in *green* indicates the ‘minimally impacted catchment zone’ (MICZ). **d** Map depicting the locations of the eight study sites, with inverted *red triangles* designating sites grouped as PICZ, and *green triangles* depicting sites grouped as MICZ. The *blue arrow*, lower left, indicates the location of the Arkansas River

Unconventional natural gas (UNG) extraction has been promoted as a potential fuel source in North America, as well as a bridge to a cleaner energy economy [1]. It is now ongoing in over 30 states, particularly those containing appropriate geologic ‘plays,’ i.e., geographic areas that contain fine-grained sedimentary rock with an appropriate clay-to-silt particle size. In North America, these include: Bakken (ND), Barnett (TX), Haynesville (LA), Fayetteville (AR), Antrim (MI), Woodford (OK), Green River (WY), Denver (CO), Marcellus and Utica (PA, OH, WV) [2] (Fig. 1a). Shale gas is termed ‘unconventional’ in that it is trapped in strata with low porosity and permeability and requires additional extraction processes beyond those normally employed in more traditional petroleum exploitations.

UNG extraction is initiated by drilling downward then horizontally into shale strata, followed by injection of 8000–50,000 m^3^ of pressurized local groundwater to fracture shale and release trapped hydrocarbons, a process termed ’fracking’ [3]. The injected water contains numerous chemical additives [4, 5] as well as ‘proppants’ (i.e., sand/silica) that lodge into fractures, allowing oil and gas to flow outward as fluid pressure subsides. Of the injected water, less than half is quickly returned to the surface (i.e., as flowback), whereas the majority (i.e., produced water) lingers underground and is slowly mobilized as gas is removed [5].

The fracking process can generate numerous environmental impacts [6], the majority of which stem from poor well integrity, improper wastewater disposal, and surface spills [3, 7], with the latter either anthropogenic or environmental (i.e., due to rainwater and/or storm flooding). Of serious concern are those that transport toxic chemicals into surface and ground water [8], with contamination directly correlated to the proximity of the drill site [9]. Impacts are most often gauged by monitoring ‘indicator species’ i.e., organisms whose presence, absence, or abundance can reflect a specific environmental condition [10], particularly in the context of adaptive stream management.

Biofilm communities in streams (*sensu lato*) are composed of sessile organisms on substrata [11] and thus have an intimate contact with, and long-term exposure to flowing waters. They provide a matrix within which fundamental ecosystem processes occur [12] and, as such, are functionally employed as bioindicators. For example, the Cyanobacterial component of biofilm can contribute >80% of the primary production in a system [13], whereas other biofilm components such as heterotrophic bacteria employ complex metabolic pathways that can quickly remediate harmful substances [14]. The composition of biofilm is radically transformed by alterations in stream conditions [15], with deterioration directly impacting the aquatic food base, such that ramifications are quickly translated into higher trophic levels [16]. Although biofilm communities play a major role in the dynamics of stream ecosystems, they have been traditionally difficult to monitor, due largely to a time-consuming process of optical identification coupled with an inability to initiate and/or sustain laboratory cultures for identification [17].

Molecular advances have now largely ameliorated these issues by facilitating identification and quantification of bacterial constituents in the biofilm community. From this, a much broader perspective on stream metabolism can be developed, in that numerous concurrent samples can be rapidly, simultaneously, and accurately characterized. For example, microbial traits are not only conserved in a phylogenetic context but also linked across clades through biochemical and genetic complexities. Important ecological traits such as pH- and salinity preferences are not only characteristic in a phylogenetic sense but also drive stream metabolism and fulfill ecosystem services [18].

Genomic approaches that characterize microbial communities are also utilized to interpret their dynamics. Here, the 16S ribosomal RNA region has been the molecular marker of choice, as it contains both conserved and hyper-variable regions that are well suited for phylogenetic analyses. Furthermore, the advent of high-throughput DNA sequencing technologies has improved accuracy and reduced costs [17], making community characterization an attractive procedure with which to gauge ecosystem health.

A molecular genetic approach was utilized in the current study to assay biofilm communities of selected streams within a 932-km^2^ region of Fayetteville shale located in the Boston Mountains of northwest Arkansas (Fig. 1b, c). The topography of this region is a limestone-based karst, with numerous emergent ground and spring-fed streams. Previous studies have assessed the potential impacts of fracking in these streams by focusing on either stream metabolism [19] or the presence/absence of aquatic insects as bioindicator species [20].

The objectives of this study were to characterize and compare the biofilm communities at sampling sites a priori characterized by fracking impacts. These sites were first evaluated across a series of abiotic and anthropogenic factors, then compared and contrasted using univariate and multivariate statistical approaches. Our results could then be evaluated against biofilm communities recorded within other shale play studies, as well as those utilizing non-microbial indicators within the Fayetteville shale [19, 20]. In addition to assaying for potential effects of fracking on stream biofilm communities, other potential anthropogenic effects that drive biofilm communities such as agriculture, silviculture, urbanization, etc., were also considered so as to guide the adaptive management of regional streams. This, in turn, provides broader insights into the manner by which the functioning of stream ecosystem can vary locally and regionally with regard to anthropogenic land manipulations, and nationally with regard to fossil fuel extraction. It also allowed us the opportunity to test if potential fracking effects could be parsed from those engendered by other anthropogenic activities.

## Results

### Biofilm collection

To understand the potential effects of fracking on stream microbial communities, we collected biofilm at eight stream sites. We grouped our sampling sites using two parameters that denoted their proximity to UNG wells. These were ‘inverse flow length’ (IFL) and ‘well density.’ Four sampling locations quite distant from UNG wells were allocated as MICZ-sites (i.e., ‘minimally-impacted catchment zone;’ = Group 1), whereas four that were significantly proximal to UNG wells were defined PICZ-sites (i.e., ‘potentially-impacted catchment zone;’ = Group 2). For easier reference, MICZ-sites and PICZ-sites are listed with an affiliated letter (i.e., A-D) that designates sampling locations in each Group (Table 1).

**Table 1:** Study sites (Sites) characterized by unconventional natural gas (UNG) activities within 1 km^2^ catchment radius

Sites	Density	IFL	Group
A = Rock creek	0.12	0.18	1
B = Driver creek	0.00	0.00	1
C = Cedar creek	0.04	0.00	1
D = Sis hollow	0.00	0.00	1
A = East fork	2.32	2.35	2
B = Sunnyside creek	3.64	0.31	2
C = Hogans creek	1.77	1.7	2
D = Black fork	0.69	1.3	2
*F*-value	11.30	10.17	
Probability	0.015^a^	0.019^a^	

Sites are geographically depicted in Fig. 1; Density is the number of unconventional natural gas (UNG) wells within a km^2^ of each site; Inverse Flow Length (IFL) represents the length of flow from each well to the stream channel, corrected for slope, and calculated for wells upstream of each sampling location using the flow length tool in ArcGIS [19]. The inverse of each flow length was summed across all wells for each catchment area such that wells more proximal had a higher value and thus a greater potential effect; Group is based on threshold values of > =0.25 wells/km^2^ and IFL >0.05, with Group 1 indicating presence within a ‘minimally impacted catchment zone’ (=MICZ), whereas Group 2 are within a ‘potentially impacted catchment zone’ (PICZ) with greater density of, and proximimty to, UNG wells; *F*-value is the F-statistic recorded in a 1-way analysis of variance (ANOVA) by Group [i.e., = MICZ (1) versus PICZ (2)] as derived in R [41]. Probability represents the statistical significance of each *F*-value as determined by Bonferroni adjusted alpha = 0.025, with significance indicated by an^a^

For each stream site, we collected two biofilm samples (one from the downstream and one from the upstream boundaries of the pool), extracted DNA, and used Illumina sequencing to evaluate a 16S rDNA molecular marker that delimits representative biofilm communities. The biofilm samples (2/site; *N* = 16) averaged 153 mg/sample (wet weight), with significantly greater amounts from downstream sections of pools as compared to those upstream (average lower = 174.4 mg, average upper = 131.1 mg; F = 7.09, *P* < 0.011, one-way ANOVA [21]). DNA concentration per sample averaged 39.8 ng/μl and did not differ by site or pool location (results not shown).

### Univariate analyses of site, hydrology, land use, and stream chemistry

We characterized ten variables at each site so as to determine whether our designated Groups differed with regard to environmental or anthropogenic factors that could, in turn, affect microbial communities. Four stream morphology variables (i.e., ‘elevation,’ ‘stream order,’ ‘%-slope,’ and ‘watershed area’) were non-significant by Group at the Bonferroni-adjusted *P*-value (results not shown). With regard to land use characteristics, MICZ-sites reflected significantly greater ‘%-forested area’ and significantly less ‘%-pasture’ (Table 2). The two Groups did not differ significantly with regard to ‘%-urban area’ at the Bonferroni-adjusted probability. In the stream chemistry analyses, PICZ-sites showed significantly greater mean values for ‘total nitrogen’ and ‘total phosphorus’ than did MICZ-sites (Table 3), suggesting more nutrient-rich catchments. Elevated values for ‘stream conductivity’ did not differ by Group at the adjusted Bonferroni-probability level.

**Table 2:** Land use characterization for Location (sampling sites) and Group (sites grouped in Table 1)

Location	Forest	Pasture	Urban	Group
A = Rock creek	1.22	0.04	0.01	1
B = Driver creek	1.29	0.02	0.01	1
C = Cedar creek	1.10	0.09	0.01	1
D = Sis hollow	0.94	0.14	0.01	1
A = East fork	0.69	0.24	0.02	2
B = Sunnyside creek	0.51	0.41	0.01	2
C = Hogans creek	0.82	0.23	0.03	2
D = Black fork	0.40	0.52	0.02	2
*F*-value	19.45	13.66	6.00	
Probability	0.005^a^	0.010^a^	0.050	

Sites are geographically depicted in Fig. 1; Allocation of sites to Group is provided in Table 1; Sites labeled as Group 1 are within a ‘minimally impacted catchment zone’ (=MICZ), whereas sites labeled as Group 2 are within a ‘potentially impacted catchment zone’ (PICZ) that contains a significantly greater density of unconventional natural gas (UNG) wells; Forest, Pasture, and Urban represent arcsin transformed values originally recorded as percentage within a 1 km^2^ radius of the catchment area; *F*-value is the F-statistic recorded in a 1-way analysis of variance (ANOVA) by Group [i.e., = MICZ (1) versus PICZ (2)] as derived in R [41]; Probability represents statistical significance of each *F*-value determined by Bonferroni adjusted alpha = 0.017, with significance indicated by an^a^

**Table 3:** Water chemistry for each Site (sampling location) and Group (sites group in Table 1)

Site	Tot-N	Tot-Ph	Conductivity	Group
A = Rock creek	0.21	0.014	0.014	1
B = Driver creek	0.07	0.012	0.012	1
C = Cedar creek	0.07	0.01	0.01	1
D = Sis hollow	0.07	0.01	0.01	1
A = East fork	0.3	0.032	0.032	2
B = Sunnyside creek	0.72	0.032	0.032	2
C = Hogans creek	0.86	0.016	0.016	2
D = Black fork	0.41	0.038	0.038	2
*F*-value	11.93	13.99	6.49	
Probability	0.014^a^	0.010^a^	0.043	

Sites are geographically depicted in Fig. 1; Allocation of sites to Group is provided in Table 1; Total nitrogen (Tot-N), total Phosphorus (Tot-Ph), and Conductivity values were originally recorded as μg/L (Tot-N and Tot-Ph) and millisieverts/cm (Conductivity) but have been log10-transformed; *F*-value is the F-statistic recorded in a 1-way analysis of variance (ANOVA) by Group [i.e., = MICZ (1) versus PICZ (2)] as derived in R [41]. Probability represents the statistical significance of each *F*-value as determined by Bonferroni adjusted alpha = 0.017, with significance indicated by an^a^

### Microbial community composition

We performed Illumina sequencing of a 16S rDNA marker as a means of identifying and quantifying microbial biofilm communities at each site. De-replication (i.e., merging of identical reads) condensed the data by 89% [i.e., from 761,914 reads into a unique set of 83,441 OTUs (operational taxonomic units)]. Elimination of singletons (i.e., OTUs that occurred but once) further reduced the total to 48,802 (a 41.5% reduction). The removal of chimeric sequences (i.e., hybrid sequences consisting of multiple OTUs) eliminated an additional 3753 (7.7%). A comparison of sequences against a reference database excluded an additional 50 (0.1%), and alignment with the core set database [22] removed an additional 345, yielding 6965 unique OTUs as a final total.

**Fig. 2:**
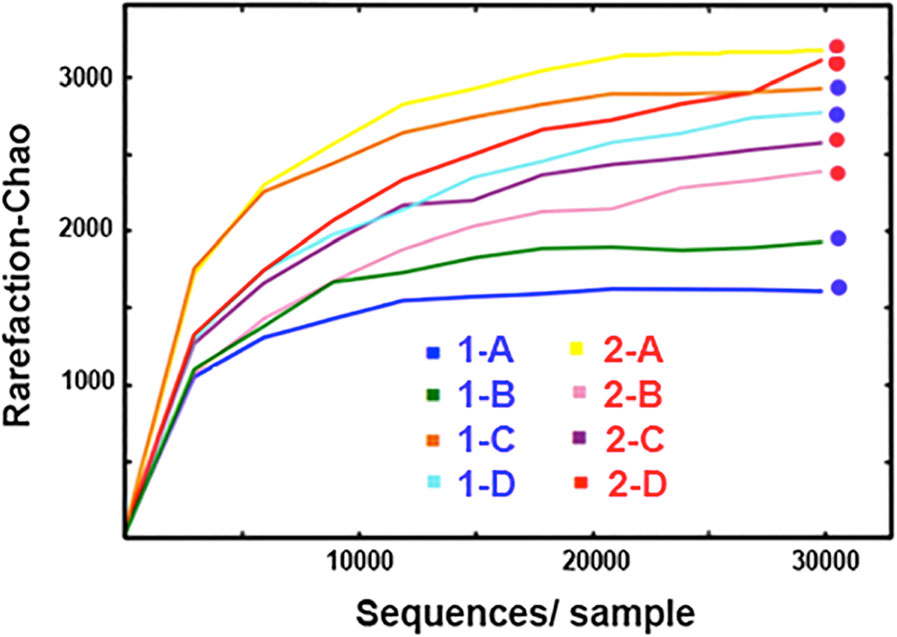
Graph depicting the number of 16S ribosomal DNA sequences generated for each of the eight study sites located in the Fayetteville shale of north-central Arkansas (X-axis) plotted according to their rarefaction scores (Chao statistic, Y-axis) as generated by the program QIIME [40]. Color of the rarefaction curve indicates study site, dots at terminus reflects ‘potentially impacted catchment zones’ (=PICZ) in *red*, or ‘minimally impacted catchment zone’ (=MICZ) in *blue*. PICZ-sites have significantly greater density of unconventional natural gas (UNG) well sites

We generated rarefaction curves that estimated alpha-diversity for each site to determine whether depth of sampling and sequencing were sufficient to adequately capture microbial community diversity. These curves approached horizontal asymptotes when plotted against number of sequence reads, suggesting sufficient sequencing depth (Fig. 2). A total of 46 phyla were represented, with 24 of these found across all samples. Average per sample = 36 (range = 32–39), with several phyla dominating across all samples: Cyanobacteria (37.4%); Proteobacteria (31.7%); Bacteroidetes (7.6%); Planctomycetes (5.3%); and Actinobacteria (4%) [21].

**Fig. 3:**
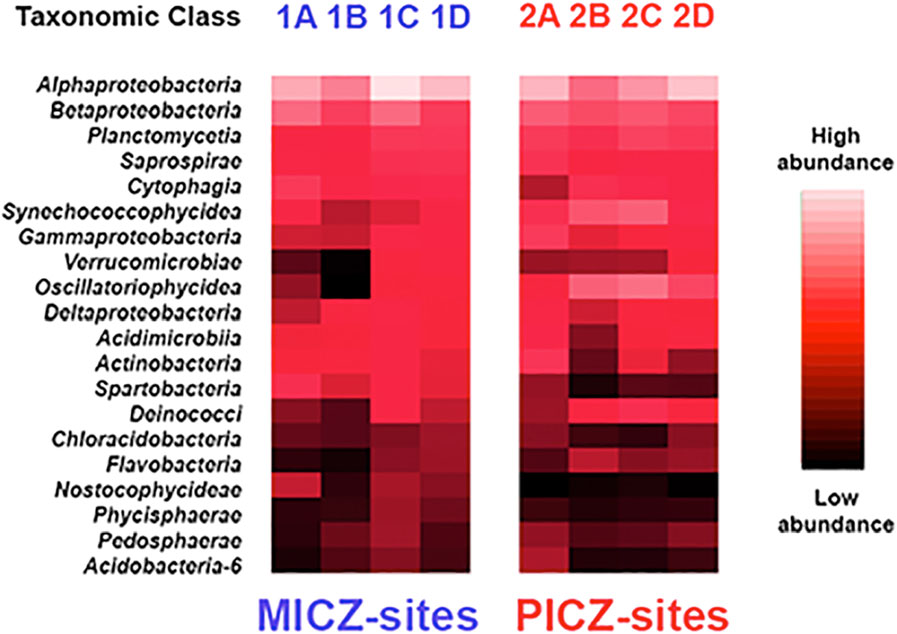
Heat map reflecting abundance of the 20-most abundant bacterial classes across the eight study sites located in the Fayetteville shale of north-central Arkansas. Columns represent study sites (X-axis) and rows are bacterial classes. The heat map was generated by the program QIIME [40] with intensities of colors (=heat) reflecting abundances as depicted by the scale to the right of the map. Study sites within ‘minimally impacted catchment zones’ (MICZ) are on the left (1-A through 1-D), whereas sites within ‘potentially impacted catchment zones’ (=PICZ) are on the right (2-A through 2-D). PICZ-sites have significantly greater density of unconventional natural gas (UNG) well sites

A total of 141 microbial classes were also represented, with 54 found across all samples. Those with average abundance >2% (*N* = 20) are presented in Fig. 3. Of these, Alphaproteobacteria was the most dominant, averaging 18.9% across samples, with Betaproteobacteria averaging 8.4%. A total of 310 genera were subsequently identified, with 116 (37%) identified across all sites and 297 (95.8%) found at ≥ 4 sites [21].

### Univariate analyses of biofilm communities

We performed univariate analyses to determine whether microbial community diversity and/or individual membership varied across MICZ and PICZ sites. Values for Shannon entropy, evenness, and number of OTUs/site did not differ significantly by Group [21], suggesting in turn that differences between groups did not broadly affect microbial diversity. The top five most abundant and the bottom three least abundant bacterial classes (Fig. 3) did not differ significantly when compared by Group [21]. We did observe differences between Groups for four other bacterial classes: the 6th (Synechoccophycideae: F_(1,6)_ = 8.24, *P* < 0.028); the 9th (Oscillatoriophycideae: F_(1,6)_ = 9.36, *P* < 0.022); 13th (Spartobacteria F_(1,6)_ = 6.36, *p* < 0.045); and 17th (Nostocophycideae: F_(1,6)_ = 14.23, *p* < 0.009), with only the latter significant at an adjusted Bonferroni-value (Table 4). Synechoccophycideae and Oscillatoriophycideae are Cyanobacteria (=primary producers), and each was more prevalent at PICZ-sites, whereas Spartobacteria and Nostocophycideae were most prevalent at MICZ-sites (Fig. 3).

**Table 4:** Four dominant microbial classes found at study sites (Site) and analyzed by Group

Site	Sparto	Synecho	Oscillato	Nostoc	Group
A = Rock creek	0.0261	0.0162	0.0050	0.0105	1
B = Driver creek	0.0075	0.0063	0.0001	0.0026	1
C = Cedar creek	0.0158	0.0076	0.0086	0.0089	1
D = Sis hollow	0.0078	0.0163	0.0105	0.0073	1
A = East fork	0.0051	0.0269	0.0125	0.0001	2
B = Sunnyside creek	0.0031	0.0636	0.0766	0.0011	2
C = Hogans creek	0.0011	0.0549	0.0638	0.0017	2
D = Black fork	0.0029	0.0223	0.0443	0.0003	2
*F*-value	6.36	8.24	9.36	14.13	
Probability	0.045	0.028	0.022	0.009^a^	

Sites are geographically depicted in Fig. 1; Allocation of sites to Group is provided in Table 1; Sparto = Bacterial class Spartobacteria, Synecho = Synechococcophycideae, Oscillato = Oscillatoriophycideae, and Nostoc = Nostocophycideae, with values representing arcsin-transformed percentages of abundance (Fig. 3); *F*-value is the F-statistic recorded in a 1-way analysis of variance (ANOVA) by Group (i.e., = MICZ versus PICZ) as derived in R [41]. Probability represents the statistical significance of each *F*-value at Bonferroni adjusted alpha = 0.017, with significance indicated by an^a^

The class Synechoccophycideae was represented by six genera, listed in descending abundance as: *Arthronema, Acaryochloris, Leptolyngbya, Pseudanabaena, Paulinella,* and *Synechococus.* In turn, seven genera composed the class Oscillatoriophycideae: *Microcystis, Chroococcus, Cyanobacterium, Chroococciddoipsis, Phoridium, and Planktothrix. Microcystis* was particularly elevated at PICZ-site 2-D (at 5.54%) [21]. Of the five Spartobacteria genera, two were identified as *Xiphinematobacter* and *Chthoniobacter* (family Chthoniobacteraceae), while the remaining three were not identified to genus. The implications with regard to the abundances of these microbial classes and genera between Groups are discussed below.

### Multivariate comparisons among group

**Fig. 4:**
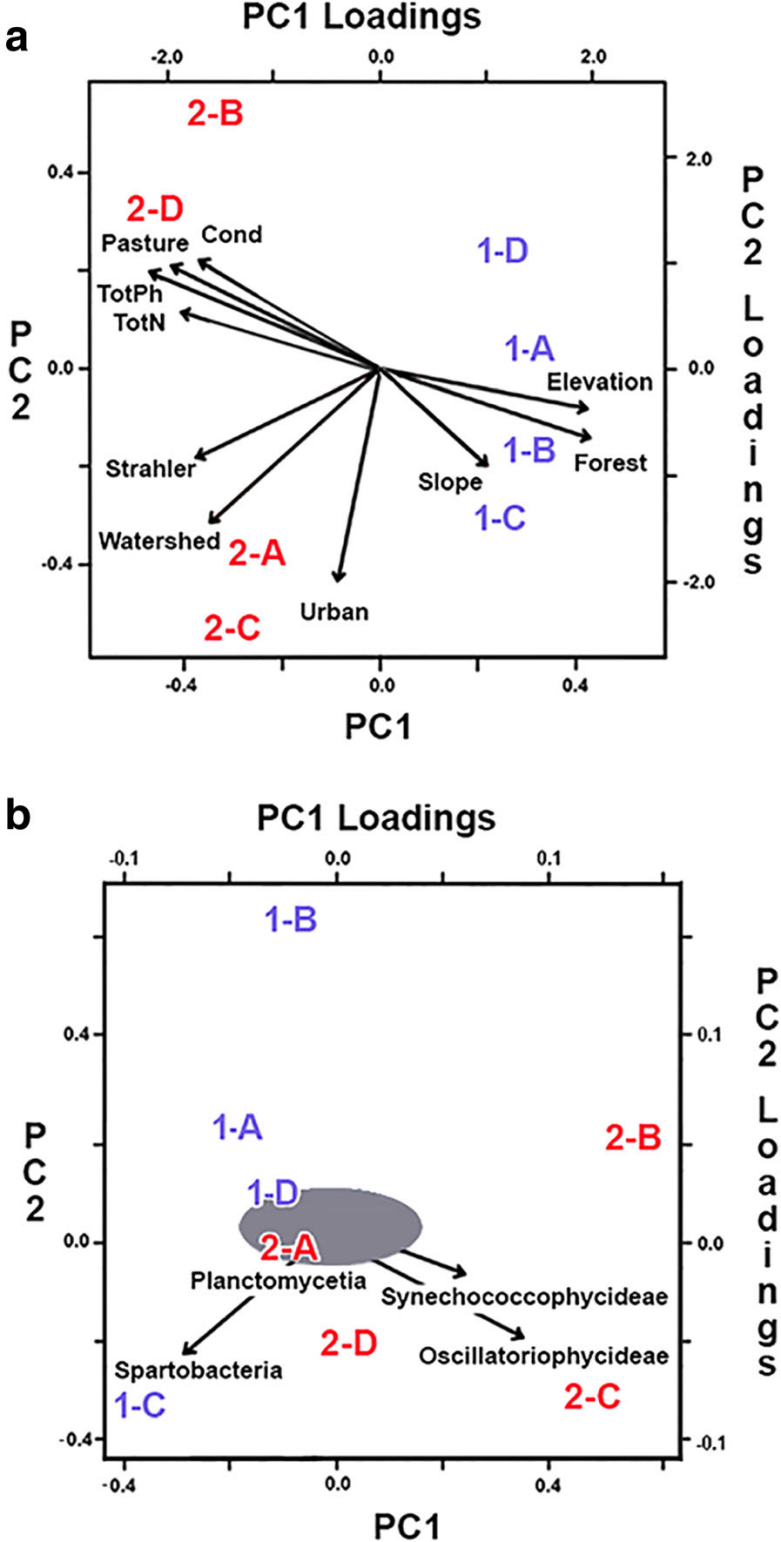
**a** Results of a biplot analysis where the first two principal components depict relationships among the eight sites in the Fayetteville shale of north-central Arkansas versus principal component loadings for a suite of ten environmental variables in three defined categories (i.e., stream morphology, land use, and water chemistry) using library “prcomp” in R [41]. Sites in *red* text are within a ‘potentially impacted catchment zone’ (=PICZ) that signifies a significantly greater density of unconventional natural gas (UNG) well sites, whereas those in *blue* text are found within a ‘minimally impacted catchment zone’ (MICZ). Variables in the biplot are represented as vectors, and the angle at their origin reflects pairwise correlations (i.e., the more acute the angle, the greater the correlation). TotPh = Total Phosphorus, TotN = Total Nitrogen, Cond = Conductance, Strahler = Stream order. **b** Results of a biplot analysis in which the first two principal components reflect relationships among the eight sites in the Fayetteville shale of north-central Arkansas versus principal component loadings for the 20-most abundant bacterial classes, where densities are represented as arcsin-transformed percentages

In Fig. 4a, a bi-plot depicts relationships within and between Groups based upon the first two principle components (PCs) of the stream morphology, anthropogenic land use, and water chemistry variables. Sites are identified according to Group (number) and Site (letter) with MICZ-sites in blue (1-A through 1-D), and PICZ-sites in red (2-A through 2-D), respectively (per Table 1). PC-1 accommodated 60% of the variation in the data, and PC-2 absorbed an additional 17% (77% total). MICZ-sites clustered to the positive (right) side of PC-1 with congruent loadings for ‘slope,’ ‘elevation,’ and ‘%-forest.’ Separation on PC-2 was more prominent for MICZ-sites, largely due to the negative values that associated sites 1-B and 1-C with ‘%-forest’ and ‘slope.’ On the positive side of PC-2, MICZ-sites 1-A and 1-D were and allied with ‘elevation.’

PICZ-sites grouped instead to the far left of the PC-1 axis, quite distinct from MICZ-sites. They still separated into quite distinct pairs, with sites 2-A and 2-C on the negative side of this axis and consistent with vectors depicting ‘watershed’ size and ‘%-urban.’ PICZ-sites 2-B and 2-D fell more distant on the positive side of the PC-2 axis, and in alliance with vectors depicting ‘conductance,’ ‘total nitrogen’, total phosphorus,’ and ‘%-pasture.’ The acute angles of these four vectors reflected their close correlation. In this regard, PICZ-site 2-D was more strongly affected than 2-B. Scores on PC-1 differed significantly by Group (*P* < 0.003; results not shown), whereas those for PC-2 did not.

In Fig. 4b, a second biplot depicted relationships within and between Groups, but in relation to the composition of their bacterial communities, with MICZ-sites in blue and PICZ-sites in red (as above). PC-1 accommodated 58% of the variation in the data, and PC-2 absorbed an additional 25% (83% total). Of the 20 bacterial classes evaluated, 16 clustered quite closely with one another and were represented by an ellipse in the plot. Four bacterial classes clearly separated from the ellipse, with arrows designating the magnitude and direction of their trajectories. PICZ-sites 2-B, 2-C, and 2-D aligned with vectors depicting classes Synechoccophycideae and Oscillatoriophycideae, whereas site 2-A grouped within the ellipse. MICZ-site 1-C was well separated and in conjunction with the class Spartobacteria, whereas class Planctomycetia separated but little from the ellipse. MICZ-site 1-D fell at the edge of the ellipse, but sites 1-B and 1-A were more distant, with 1-B particularly so.

Linear iscriminant analysis Effect Size (LEfSe) analyses corroborated much of the above by delineating 20 OTUs with an LDA score > 2.7. Subclasses Oscillatoriophycideae and Synechococcophycideae (Cyanobacteria), and Roseiflexales (a filamentous bacteria often found with Synechococcophycideae and deemed tolerant of eutrophication and/or poor water quality) were abundant at PICZ-sites. Bioindicators of healthy streams [i.e., families Rhodocyclaceae (Proteobacteria), Stigonematales (Cyanobacteria), and Rivulariaceae (Cyanobacteria)] were abundant at MICZ-sites, as were ‘negative bioindicators’ (*N* = 5; primarily Spartobacteria) whose abundances covary negatively with particular impacts such as elevated pH or salt concentrations.

## Discussion

During the past decade, shale resources have been heavily developed in the United States, an industry that will steadily increase over the next several years [10]. The majority of environmental impacts that stem from these activities parallel those recorded for traditional petroleum-extraction, and as such can be predictably monitored [23]. Others are instead UNG-specific, such as poor well integrity and accidental wastewater release, and are compounded by the geographic distribution of shale plays across the continent [24] (Fig. 1a). Environmental risks associated with UNG are hence more difficult to predict and to track, in that sufficient data regarding their breadth and depth have yet to accumulate. This, in turn, delays the designation of appropriate environmental policies that would otherwise provide for their regulation [25, 26].

Research activities that evaluate these impacts are ongoing in the Fayetteville Shale of northwest Arkansas [19, 20] (Fig. 1b, c), and have now been expanded so as to encompass biofilm communities as biological indicators of study catchments (this study). The composition of microbial communities reflects sensitivity and exposure of these catchments to anthropogenic activities [14], such as urbanization, deforestation, agricultural development, habitat fragmentation, and others [27], including UNG-extraction. It is of interest to potentially parse these situations according to the manner by which they drive stream microbial diversity. Similarly, ecosystem processes are also driven by hydrology, stream gradient, stream order, and stream chemistry (among others), and these also modulate the composition of biofilm communities [15]. Given this, we first tested (and rejected) the hypothesis that environmental variability was similar among our minimally impacted (MICZ) versus potentially impacted (PICZ) study sites.

### Ecological variation among study sites

Instead, we found significant differences among several test variables, as evaluated by Group. For example, MICZ-sites reflected catchments with significantly greater ‘%-forested’ area, but significantly less ‘%-pasture’ (Table 2). Of interest is the fact that several other variables showed elevated but not significantly different values, as gauged by the Bonferroni-corrected probability value for multiple comparisons. We comment on this situation below.

Significant environmental differences between the two groups were also noted when multivariate analyses incorporated the ten variables across the three categories. Sites separated along PC-1, with strong positive (MICZ) and negative (PICZ) loadings manifested according to stream morphology, anthropogenic land use, and water chemistry (Fig. 4a). There was also considerably more variance among PICZ-sites, with paired catchments (i.e., 2-A/2-C and 2-D/ 2-B) well separated on PC-2. Somewhat surprisingly, these sites also showed a strong and concerted response to those variables not deemed significant in the univariate analyses. MICZ-sites displayed much less variability, yet were similarly separated on PC-2 according to a composite of variables that were significant (i.e., ‘%-forest’) and non-significant (i.e., ‘slope’ and ‘elevation’).

The univariate statistics provided differentiation by Group according to individual variables evaluated singly whereas the multivariate analyses provided broader patterns much more interpretable at the ecosystem level, yet not apparent from the separate univariate analyses. This was due largely to the reduced degrees of freedom in the univariate analyses, as constrained by small sample sizes parsed between groups and gauged with Bonferroni-adjusted probabilities. Although our multivariate analyses did not provide statistical probabilities within a hypothesis-testing framework, they more easily depicted the disparity within- and among-Groups, as promoted by watershed, land use, and water chemistry.

### The variability in biofilm communities among study sites

Having established the environmental context for our sampling sites by Group, we could then contrast their biofilm communities (Fig. 4b). Groups again separated in multivariate space, albeit less distinctively and with a greater spread among PICZ-sites along PC-1. PICZ-sites also exhibited less variation than did MICZ-sites along PC-2. Clearly, microbial composition varies both among- and within-sites, but with different microbial taxa driving this result in each Group.

For example, Spartobacteria (non-significant in the 1-way ANOVA) clearly associated with MICZ-site 1-C, and differentiated it from all others, whereas site 1-B (associated with 1-C in Fig. 4a) was arrayed quite distantly from other MICZ-sites on PC-2. Furthermore, one site from each Group (i.e., 1-D/ 2-A) fell close to the origin of the PC-axes, suggesting a low overall diversity in their microbial composition (results not shown).

These differences indicate a disparity between biofilm community composition and environmental variability among sites, a result that parallels those from other studies. For instance, microbial assemblages will often group according to historical (i.e., phylogenetic) events [28], but also in response to more contemporary biochemical conditions associated with streams [29]. In this study, numerous factors (per Tables 1, 2 and 3) obviously impact the relationship between biofilm communities and their environment. This result confounds any ‘cause-and-effect’ scenarios for the observed patterns. However, we draw two strong conclusions from the multivariate analyses: PIZC-sites display greater ecological variability within the environmental matrix (Fig. 4a), yet are much less variable when embedded within the biofilm community matrix (Fig. 4b).

An alternate approach would be to contrast our results with those from studies in other shale plays that employed biofilm communities as bioindicators of fracking. Yet many of the latter are tangential to the present study, in that they examined biofilm communities in either flowback [30] or produced waters [30, 31]. Few evaluated stream catchments into which groundwater from fracked sites would eventually percolate, as herein. One study that did so evaluated headwater streams in the Marcellus shale (PA) (Fig. 1a), and found significantly lower species richness and evenness values at sites impacted by fracking [24]. Several of these sites also contained an abundance of bacterial OTUs that correlated positively with decreasing pH, suggesting more acidic stream environments. The diversity of carbon sources available in a stream promotes the functioning of its biofilm communities, and as diversity decreases, so does the community [32]. In this sense, a reduction in carbon sources would be an ecological explanation for the observed reduction in species richness at these sites, although this was not stated as such.

### The richness and evenness of species within biofilm communities

In our study, the species richness, evenness, and number of OTUs in biofilm communities were not significantly different when compared between MICZ- and PICZ-sites. Yet such comparisons often mask the interactions among OTUs within these communities. For example, a decrease in abundance of some taxa can also stimulate growth in others normally more rare, a situation that would promote rather than depress evenness [29, 32]. Those streams with lowest values for evenness in each of our Groups [i.e. 1-B and 2-D; 21] may indeed reflect this consideration. For example, 1-B is a headwater stream (stream order = 1) with the greatest ‘%-forest’ in the study (=96%) both of these environmental aspects would promote deposition of leaf litter into the stream that, in turn, must be decomposed. This similarly constrains the biofilm community.

The two least diverse streams in an ecological sense (i.e., 1-A and 1-B) also had low numbers of OTUs [21], again suggesting the potential for a reduction in available nutrients [19]. In a similar vein, PICZ-site 2-D had the highest value for ‘%-pasture’ in the study, and was also associated with elevated levels of available phosphates and nitrates (Fig. 4a), both of which can promote a few dominant species. This was represented at PICZ-sites by the elevated abundances of two Cyanobacterial classes (i.e., Synechococcophycideae and Oscillatoriophycideae). Cyanobacteria are primary producers that seemingly track the significantly elevated levels of nitrogen and phosphorus found in these streams.

A second but related limitation with regard to species richness and evenness is the strong competition among bacteria and hyphomycetes (stream fungi), as promoted by the reduction in dissolved organic matter (DOM) [27]. Dissolved nitrogen primarily exists as nitrates within ground and surface waters, and must be transformed by microbes before entering into and moving through the ecosystem. This, in turn, could promote bacterial OTUs more strongly competitive, at the expense of those less competitive, a situation that would also constrain biofilm community diversity. In addition, and as a second consideration, elevated nitrogen levels are often associated with UNG well sites [19].

Additionally, the removal of pollutants can, paradoxically, also reduce microbial diversity and evenness [33], suggesting (as above) that external sources of carbon can promote the development of OTUs normally more rare. These caveats, in turn, provide numerous potential corollaries to explain the low values for evenness at sites, particularly when carbon sources have become more limited due to fracking [24, 32].

### Biofilm communities as bioindicators

The function of many bacterial lineages is not well understood at the ecosystem level, despite their abundances in soil and aquatic systems, and this in turn makes it more difficult to ascertain their status as potential bioindicators. Despite this, general functions are indeed assignable to some clades. Many Synechococcophycideae, for example, employ unique metabolic pathways that allow them to persist in highly acidic environments such as volcanic seeps. The Oscillatoriophycideae is an equally diverse clade that can also serve a bioindicator for organic pollutants. For example, *Microcystis* (a genus of Oscillatoriophycideae) is abundant at PICZ-sites, and its presence may point to the presence of elevated polycyclic aromatic hydrocarbons (PAHs) that in turn promote its growth [34].

In addition, the genomes of aquatic Spartobacteria encode for a diversity of glycoside hydrolases that are employed in the degradation of complex carbohydrates [35]. This physiological aspect also explains its common co-occurrence with Cyanobacteria, in that the former metabolizes the complex carbohydrates produced by the latter [12]. Spartobacteria should thus positively correlate with Cyanobacteria at PICZ-sites, but was instead found to be significantly reduced. Brine contamination is a well-known fracking by-product, and it continues to be pulled upwards from deeper strata long after drilling has subsided [36]. In addition, PICZ-sites also reflected greater conductance in their water chemistry. Spartobacteria has a pronounced intolerance for salt, and these environmental conditions at PICZ-sites would impede its expected proliferation.

In an attempt to gain a more comprehensive perspective, we can also contrast results from this study with those from earlier studies at the same sites. For example, UNG development had a definite impact on stream macroinvertebrate communities, with short-lived generalists being more abundant at those sites [20]. Yet these effects were difficult to parse across specific taxa, or to specifically associate with the benthic habitat found at PICZ-site.

A second study [19] found increased primary production and eutrophication at sites impacted by UNG activities, and this was interpreted as a potential response to the enhanced levels of nitrogen these sites displayed. Our data support these conclusions in that two classes of Cyanobacteria were clearly more abundant at impacted sites, suggesting the presence of an environment that is beneficial for primary production. Our statistical analyses also verified significant levels of ‘total nitrogen’ and ‘total phosphorus’ at these sites, as well as heightened conductance.

Overall, the observed differences between MICZ-sites and PICZ-sites may reflect the accessibility of sites chosen for UNG well construction, and as such, may add an additional consideration for the design controlled studies to gauge the effects of fracking (see Discussion).

## Conclusions

Biofilm communities have complex roles in freshwater stream metabolism, and consequently drive numerous critical processes: Primary production [12], biogeochemical cycling [17], nitrogen cycles [28], and the remediation of deleterious carbon sources [14], among many. Microbial communities are also extraordinarily diverse, composed of numerous rare OTUs, and display a rapid response to changes in temperature, pH, and stream metabolism [16]. This also provoke taxonomic turnover in stream biofilm communities as an ecosystem-scale response [29]. Given this, stream biofilm communities can be employed to only to gauge ecosystem health [28], but also its potential impacts on humankind [17]. Unfortunately, the breadth and depth of biofilm communities are also confounding factors that can limit diagnostic and taxonomic projections, particularly with regard to bioremediation.

Region specific issues also predominate [4]. For example, biofilm communities are quite sensitive to changes in land use [15]. This is important in that both the Fayetteville and Barnett shale catchments display pre-existing anthropogenic disturbances [26] that can easily confound more focused analyses regarding the impacts of UNG-activity. In addition, habitat and water chemistry data collected prior to the onset of fracking are necessary baselines from which potential impacts on both freshwater streams and their biofilm communities can be assessed. These data were lacking herein, and similarly lacking in other studies that employed biofilm communities as a means to adjudicate fracking activities [24]. As a result, the statistical analyses employed to contrast these sites were similarly limited.

Unfortunately, necessary data are often unavailable at the national level, and a mandate for their collection has not as yet been established in state or federal management plans. This, in turn, cripples the development of conservation measures that may promote the sustainability of stream ecosystems. Resource managers require these data so as to guide local development projects, and to reduce possible environmental effects particularly in light of the interactive effects produced by multiple stressors in a warming climate [12]. The evaluation of anthropogenic impacts, whether fracking or otherwise, also depends upon rigorous statistical analyses conducted in a comparative manner (as herein). This, too, is often lacking with regard to those projects that attempt to recognize and define biodiversity elements, or conserve and restore habitats.

Our data mirror similar conditions found in other systems with long-term disturbance, such as elevated conductance/lack of Spartobacteria, and elevated nitrogen/elevated Cyanobacteria, and these, in turn, suggest potential impacts from UNG wells. Our data are also confounded by pre-existing conditions such as development of pasture and the extent of urbanization, as well as naturally occurring aspects such as stream order that likewise influence the constituents of biofilm communities, and biodiversity in general. These limitations argue for an a priori selection of pre- versus post-impact study sites, in that a variety of anthropogenic endeavors can drive biofilm communities in concurrent directions and it is difficult if not impossible to separate these effects a posteriori. The complexities of anthropogenic/environmental interactions also necessitate the development of a rigorous statistical framework, one within which variability can be tested among- and between-groups. This study provides a set of guidelines with regard to study design that can avoid the former, while establishing a strong statistical framework for the latter.

## Methods

### Sampling sites and environmental data for catchments

Eight sites from an ongoing stream ecology project [19, 20] (Fig. 1b, c, d) were assigned to ‘Group’ using two parameters that relate to UNG-well activity: ‘well density’ and ‘inverse flow length’ (IFL). ‘Well density’ is defined as the number of UNG well sites within a 1-km^2^ radius (=catchment area), whereas ‘IFL’ represents the length of flow from each well site to the stream channel, corrected for slope, and calculated for wells upstream of each sampling location via the flow length tool in the ‘Spatial Analyst Toolkit’ of ArcGIS [19]. The inverse of each flow length was summed across all well sites for each catchment area, such that wells more proximal had a higher value that corresponded to a greater potential effect. Sites with an IFL < 0.25 and Well Density (no./km2) < 0.5 were scored as ‘1,’ and designated as a ‘minimally-impacted catchment zone’ (MICZ), whereas those with an IFL ≥ 0.25 and a Well Density ≥ 0.5 were scored as ‘2’ and grouped as a ‘potentially-impacted catchment zone’ (PICZ).

We characterized ten variables distributed across three categories at each site so as to ascertain if designated Groups differed with regard to environmental or anthropogenic factors that could, in turn, affect microbial communities. The first category related to stream morphology and employed four variables (i.e., ‘elevation,’ ‘stream order,’ ‘%-slope,’ and ‘watershed area’). The second utilized three variables that summarized anthropogenic land use (i.e., ‘%-forest,’ ‘%-pasture,’ and ‘%-urban’). The third recorded three water-chemistry parameters (i.e., ‘total nitrogen,’ ‘total phosphorus,’ and ‘conductivity’) deemed important in gauging relationships between stream metabolism and bacterial communities [19]. Abundance of nutrients was measured as μg/L, whereas dissolved salt/ions was in microSiemens (*u*S)/cm, with higher values signaling an elevated presence of ions.

### Biofilm collection, DNA extraction, and Illumina sequencing

At each site, a pool was identified peripheral to the greatest stream flow and a biofilm-covered rock was then selected at downstream (lower) and upstream (upper) boundaries and scrubbed with a sterile Nasco Whirl-Pak Speci-Sponge™. Sponges were immediately re-sealed in the sterile Whirl-Pak and placed onto dry ice for transport to the lab where they were stored at −80°C until processed. For DNA extraction, 20 ml of phosphate buffered saline solution (PBS; 137 mM NaCl, 2.7 mM KCl, 4.3 mM Na_2_HPO_4_, 1.47 mM KH_2_PO_4_, pH 7.4) was added to each sample, and the sponge squeezed manually for 5 min to suspend biofilm. Suspensions were transferred to individual centrifuge tubes and pelleted by centrifugation (8000 *g* for 20 min), with biofilm quantified via wet weight (mg). Standard laboratory protocols were used for all procedures to prevent sample contamination.

DNA from pelleted biofilm was extracted for all 16 samples (2 per site) using a MOBIO commercial kit (PowerBiofilm® DNA Isolation Kit) following manufacturer’s instructions. DNA was quantified (ng/ul) using a Qubit 2.0 Fluorometer (Invitrogen®). Extractions were subjected to PCR using primers that amplified the hyper-variable V4 region of the 16S structural subunit rRNA gene [37]. Multiplexed 16S metagenomic libraries were constructed using standard Illumina protocols, and were sequenced on an Illumina MiSeq platform. Raw Illumina reads were de-multiplexed (MiSeq Reporter software™) and downloaded from the Illumina BaseSpace® cloud.

### Bioinformatics

Sequences were trimmed to 251 bp and quality filtered at an expected error of <1% using USEARCH v8.0 [38]. A pipeline developed by the Brazilian Microbiome Project [39] was employed to correct any Illumina formatting issues for subsequent analyses in QIIME v1.7 [40]. OTUs were selected with the UCLUST method (as implemented in QIIME) and taxonomy assigned using the Greengenes 16S rRNA gene database [22], with subsequent conversion into an OTU table (QIIME).

### Univariate analyses

Prior to analyses, nine variables were transformed: Percentages (*N* = 4) were arcsin transformed to radians; areas (*N* = 1) reduced to square root; and quantitative variables (*N* = 5) transformed to log_10_. ‘Stream order’ was evaluated as recorded. Each category was test by Group using a 1-way analysis of variance in R [41], with statistical significance assigned according to Bonferroni-corrected probabilities.

Shannon entropy was computed in QIIME to gauge the number of unique bacterial taxa in each community (i.e., richness) and the evenness of their distributions, with results compared by Group using a 1-way ANOVA in R. Species richness (with repeated subsampling) was then plotted by site as rarefaction curves, so as to estimate whether sampling at each site was of sufficient depth to accurately characterize biofilm communities. Analyses were carried out with the default number of Monte-Carlo permutations (*N* = 999) at a *p*-value of 0.05. UniFrac analyses (in QIIME) were used to derive beta (or between sample) diversity estimates using both unweighted data (i.e., OTU presence/absence) and weighted (by relative abundance) [42]. To identify potential bioindicators, a heat map was generated in QIIME using the 20-most abundant taxonomic classes of bacteria. Potential bioindicators were then identified and compared by Group using a 1-Way ANOVA in R with Bonferroni-corrected probabilities.

### Multivariate analyses

A principal components analysis (PCA) was performed using a matrix of correlations among sites based on the ten variables across the three categories (i.e., stream morphology, anthropogenic land use, and stream chemistry) using library “prcomp” in R [41]. The first two principal components depicted relationships among the eight sites (i.e., PC-scores) and were contrasted against principal component loadings for the variables. Both scores and loading were visualized in a single plot (hence the term, ‘biplot’), so as to promote the interpretation of the component axes in relation to the variables. Those in the biplot were represented as vectors, and the angle at their origin(s) reflects pairwise correlations (i.e., the more acute the angle, the greater the correlation). We then compared the first six principal components by Group in R, using a 1-way ANOVA with Bonferroni-corrected probabilities.

A principal component analysis was also used to contrast densities of the 20-most abundant bacterial classes among study sites (using library “prcomp” in R [41]), with densities represented as arcsin-transformed percentages. The first two principal components depicted relationships among the eight sites (i.e., PC-scores) and were contrasted against principal component loadings for the 20-most abundant classes.

The biomarker discovery algorithm LEfSe (Linear discriminant analysis Effect Size) was used to designate potential bioindicators among biofilm communities [43]. The program employs a linear discriminant analysis (LDA) with effect size estimated by linking output to the level-6 (Kingdom to Genus) taxonomic summary in QIIME. Parameters employed were: an alpha value of 0.05 for the Kruskal-Wallis (KW) test, an LDA score threshold of >2.7, and a pairwise Group-comparison. Initially, LEfSe conducts the KW rank-sum test as a means of detecting OTUs that differed significantly in abundances between Groups. Biological significance was then investigated with the (unpaired) Wilcoxon rank-sum test. Finally, LDA was then employed to evaluate each OTU with an effect size > 2.7, and with biological indicator gauged via habitat and metabolism.
